# Risk Factors for Pandemic (H1N1) 2009 Seroconversion among Adults, Singapore, 2009

**DOI:** 10.3201/eid1708.101270

**Published:** 2011-08

**Authors:** Wei-Yen Lim, Cynthia H.J. Chen, Yi Ma, Mark I.C. Chen, Vernon J.M. Lee, Alex R. Cook, Linda W.L. Tan, Norberto Flores Tabo, Ian Barr, Lin Cui, Raymond T.P. Lin, Yee Sin Leo, Kee Seng Chia

**Affiliations:** Author affiliations: National University of Singapore, Singapore (W.-Y. Lim, C.H.J. Chen, Y. Ma, M.I.C. Chen, V.J.M. Lee, L.W.L. Tan, N. Flores Tabo, Jr., K.S. Chia);; Tan Tock Seng Hospital, Singapore (M.I.C. Chen, Y.S. Leo);; Ministry of Defence, Singapore (V.J.M. Lee);; National University of Singapore, Singapore (A.R. Cook);; World Health Organization Collaborating Centre for Reference and Research on Influenza, Melbourne, Victoria, Australia (I. Barr);; Ministry of Health, Singapore (L. Cui, R.T.P. Lin)

**Keywords:** Influenza A virus, pandemics, seroepidemiologic study, cohort analysis, pandemic (H1N1) 2009, influenza, risk factors, travel, public transport, viruses, Singapore, adults, research

## MEDSCAPE CME

Medscape, LLC is pleased to provide online continuing medical education (CME) for this journal article, allowing clinicians the opportunity to earn CME credit.

This activity has been planned and implemented in accordance with the Essential Areas and policies of the Accreditation Council for Continuing Medical Education through the joint sponsorship of Medscape, LLC and Emerging Infectious Diseases. Medscape, LLC is accredited by the ACCME to provide continuing medical education for physicians.

Medscape, LLC designates this Journal-based CME activity for a maximum of 1 *AMA PRA Category 1 Credit(s)*^TM^. Physicians should claim only the credit commensurate with the extent of their participation in the activity.

All other clinicians completing this activity will be issued a certificate of participation. To participate in this journal CME activity: (1) review the learning objectives and author disclosures; (2) study the education content; (3) take the post-test with a 70% minimum passing score and complete the evaluation at www.medscape.org/journal/eid; (4) view/print certificate.

**Release date: July 23, 2011; Expiration date: July 23, 2012**

## Learning Objectives

Upon completion of this activity, participants will be able to:

- Analyze the demographic risk factors for seroconversion for pandemic (H1N1) 2009
- Evaluate the environmental risk factors for seroconversion for pandemic (H1N1) 2009
- Design public health interventions to reduce the spread of pandemic (H1N1) 2009
- Identify laboratory markers indicating a lower risk for seroconversion for pandemic (H1N1) 2009

## MedscapeCME Editor

**Karen Foster, **Technical Writer/Editor, *Emerging Infectious Diseases. Disclosure: Karen Foster has disclosed no relevant financial relationships.*

## Medscape CME Author

**Charles P. Vega, MD**, Associate Professor; Residency Director, Department of Family Medicine, University of California, Irvine. *Disclosure: Charles P. Vega, MD, has disclosed no relevant financial relationships.*

## Authors

*Disclosures:* ***Wei-Yen Lim, MBBS; Cynthia H.J. Chen; Yi Ma; Mark I.C. Chen, PhD; Alex R. Cook, PhD; Linda W.L. Tan, BSc; Norberto Flores Tabo, Jr.; Ian Barr, PhD; Lin Cui, PhD; Raymond T.P. Lin, MBBS;*** *and* ***Kee Seng Chia, MBBS,*** *have disclosed no relevant financial relationships.* ***Vernon J.M. Lee, MBBS,*** *has disclosed the following relevant financial relationships: received a grant from GlaxoSmithKline for unrelated public health research.* ***Yee Sin Leo, MBBS,*** *has disclosed the following relevant financial relationships: served as an advisor or consultant for Sanofi-Aventis on dengue vaccine.*
