# Supplementary material for: Risk Factors for Pandemic (H1N1) 2009 Seroconversion among Adults, Singapore, 2009
Source: Emerg Infect Dis. 2011 Aug;17(8):1455–62. doi: 10.3201/eid1708.101270 (PMC3381584; doi:10.3201/eid1708.101270)
Supplement: Technical Appendix — Baseline measures of social contact and interaction, Bivariate logistic regression, and Multivariate logistic regression analysis tables. [file 10-1270-Techapp_3p.pdf]

Article DOI: 10.3201/eid1708.102170

Risk Factors for Pandemic (H1N1) 2009 Seroconversion among Adults, Singapore, 2009

Technical Appendix

Technical Appendix Table 1. Baseline measures of social contact and interaction in a cohort of 828 community-dwelling adults participating in a study of risk for pandemic (H1N1) 2009, Singapore, 2009

Measure	Seroconverted, no. (%), n = 98	Did not seroconvert, no. (%), n = 629	HR* (95% CI)†	Mean p value‡
Use of public transport in preceding 14 d				
Never	16 (11.2)	127 (88.8)	1.00	
Once or twice	10 (9.4)	96 (90.6)	0.80 (0.31–2.04)	0.631
Several times	27 (14.3)	162 (85.7)	1.39 (0.70–2.78)	0.385
Daily or almost daily	45 (15.6)	244 (84.4)	1.55 (0.81–2.94)	0.222
Use of public transport in preceding 14 d, dichotomized				
Never/once or twice, i.e., seldom	72 (15.1)	406 (84.9)	1.00	
Several times/daily or almost daily, i.e., frequent	26 (10.4)	223 (89.6)	1.61 (0.96–2.70)	0.106
Visits to mass entertainment venues in preceding 14 d				
Never	70 (12.4)	496 (87.6)	1.00	
Once or twice	24 (17.7)	112 (82.4)	1.44 (0.86–2.40)	0.213
Several times	4 (17.4)	19 (82.6)	1 (0.29–3.59)	0.675
Daily or almost daily	0	2 (100.0)	0 (0–∞)	0.493
Visits to shopping centers/markets or supermarkets in preceding 14 d				
Never	11 (16.4)	56 (83.6)	1.00	
Once or twice	28 (10.7)	234 (89.3)	0.54 (0.25–1.15)	0.147
Several times	47 (15.8)	251 (84.2)	0.84 (0.41–1.71)	0.633
Daily or almost daily	12 (12.0)	88 (88.0)	0.66 (0.27–1.60)	0.376
Visits to places of worship in preceding 14 d				
Never	49 (13.4)	318 (86.7)	1.00	
Once or twice	27 (11.6)	205 (88.4)	0.96 (0.58–1.60)	0.811
Several times	18 (19.2)	76 (80.9)	1.30 (0.69–2.43)	0.452
Daily or almost daily	4 (11.8)	30 (88.2)	0.80 (0.25–2.58)	0.706
Visits to restaurants/hawker centers/bars and clubs in preceding 14 d				
Never	18 (10.1)	160 (89.9)	1.00	
Once or twice	24 (13.7)	151 (86.3)	1.01 (0.52–1.96)	0.818
Several times	39 (17.1)	189 (82.9)	1.38 (0.77–2.47)	0.320
Daily or almost daily	17 (11.6)	129 (88.4)	0.85 (0.42–1.73)	0.664
Visits to social gatherings with ≥10 persons in preceding 14 d				
Never	69 (14.9)	393 (85.1)	1.00	
Once or twice	22 (10.7)	184 (89.3)	0.69 (0.41–1.18)	0.210
Several times	4 (7.8)	47 (92.2)	0.57 (0.21–1.58)	0.302
Daily or almost daily	3 (37.5)	5 (62.5)	0.89 (0.16–∞)	0.667

*Calculated as the mean HR of 1,000 separate analyses, where each analysis comprised 610 persons from different households; 1 person was randomly selected from households that had >1 member in the study. HR, hazard rate; CI, confidence interval.
†The means of the upper and lower 95% CIs were computed for each variable.
‡The mean of the p estimate from the 1,000 iterations was computed for each variable.

Technical Appendix Table 2. Bivariate logistic regression of sociodemographic and individual variables in a cohort of 727 community-dwelling adults, Singapore, 2009*

Characteristic	Seroconverted, n = 98	Did not seroconvert, n = 628	OR (95% CI)	p value
Age group, y, no. (%)				
20–29	21 (16.9)	103 (83.1)	1.00	
30–39	16 (14.2)	97 (85.8)	0.81 (0.40–1.64)	0.557
40–49	42 (16.2)	218 (83.8)	0.94 (0.53–1.68)	0.847
50–59	15 (9.7)	140 (90.3)	0.53 (0.26–1.07)	0.076
≥60	4 (5.3)	71 (94.7)	**0.28 (0.09–0.84)**	**0.023**
Sex, no. (%)				
F	52 (12)	380 (88)	1.00	
M	46 (15.6)	249 (84.4)	1.35 (0.88–2.07)	0.169
Ethnicity, no. (%)				
Chinese	6 (6.7)	84 (93.3)	1.00	
Malay	56 (16.9)	275 (83.1)	**2.85 (1.19–6.85)**	**0.019**
Indian	34 (11.4)	265 (88.6)	1.80 (0.73–4.43)	0.203
Other	2 (28.6)	5 (71.4)	5.60(0.89–35.16)	0.066
Dwelling type				
≤3-room public housing	21 (11.9)	156 (88.1)	1.00	
4-room public housing	51 (16.5)	259 (83.5)	1.46 (0.85–2.52)	0.172
5-room public housing, private housing	26 (10.8)	214 (89.2)	0.90 (0.49–1.66)	0.742
Smoking, no. (%)				
Current smoker	30 (20.1)	119 (79.9)	1.00	
Nonsmoker, former smoker	68 (11.8)	510 (88.2)	**0.53 (0.33–0.85)**	**0.008**
Members in household participating in study, no. (%)				
1	63 (12.7)	433 (87.3)	1.00	
2	33 (16.9)	162 (83.1)	1.40 (0.89–2.21)	0.150
3	2 (6.1)	31 (93.9)	0.44 (0.10–1.90)	0.273
4	0	3 (100)	0 (0–∞)	0.980
Self-reported previous influenza vaccination, no. (%)				
No	88 (13.3)	574 (86.7)	1.00	
Yes	10 (15.4)	55 (84.6)	1.19 (0.58–2.41)	0.638
Employment outside the home, no. (%)				
No	42 (15.2)	235 (84.8)	1.00	
Yes, without anyone at work having symptoms of ARI during study period, no. (%)	39 (11.2)	310 (88.8)	0.70 (0.44–1.12)	0.141
Yes, with anyone at work having symptoms of ARI during study period	17 (16.8)	84 (83.2)	1.13 (0.61–2.10)	0.692
Baseline antibody titer			**0.54 (0.31–0.94)**	**0.029**
Mean	0.082	0.272		
Median	0	0		
No. household members, mean				
0–4 y				
Mean	0.204	0.291	0.77 (0.52–1.15)	0.202
Median	0	0		
Range	0–3	0–3		
5–19 y				
Mean	1.57	1.22	**1.22 (1.04–1.43)**	**0.013**
Median	1.0	1		
Range	0–6	0–5		
>19 y				
Mean	3.33	3.1	1.14 (0.98–1.33)	0.096
Median	3	3		
Range	1–8	1–9		
Household contact with ARI symptoms during course of study, no. (%)				
No	72 (14.4)	428 (85.6)	1.00	
Yes	26 (11.5)	201 (88.5)	0.77 (0.48–1.24)	0.282
Travel out of Singapore during study period, no. (%)				
No	50 (10.2)	438 (89.8)	1.00	
Yes	48 (20.1)	191 (79.9)	**2.20 (1.43–3.39)**	**<0.001**
Public transport, no. (%)				
Seldom	26 (10.4)	223 (89.6)	1.00	
Frequent	72 (15.1)	406 (84.9)	1.52 (0.94–2.45)	0.085

*Participants who provided at least 1 blood sample in addition to that obtained at baseline. OR, odds ratio; CI, confidence interval; ARI, acute respiratory infection. **Boldface** indicates statistically significant association.

Technical Appendix Table 3. Multivariate logistic regression analysis of factors associated with seroconversion to pandemic (H1N1) 2009 in a cohort of community-dwelling adults, Singapore, 2009*

Characteristic	Odds ratio (95% confidence interval)	p value
Age group, y		
20–29	1.00	
30–39	1.11 (0.49–2.53)	0.799
40–49	1.16 (0.55–2.43)	0.697
50–59	0.63 (0.29–1.40)	0.258
≥60	0.34 (0.10–1.14)	0.081
Sex		
F	1.00	
M	**1.95 (1.09–3.47)**	**0.024**
Ethnicity		
Chinese	1.00	
Malay	2.41 (0.92–6.32)	0.075
Indian	2.14 (0.81–5.64)	0.126
Other	6.74 (0.93–48.61)	0.058
Dwelling type		
≤3-room public housing	1.00	
4-room public housing	1.19 (0.65–2.21)	0.571
5-room public housing, private housing	0.73 (0.37–1.47)	0.382
Smoking		
Current smoker	1.00	
Nonsmoker, former smoker	0.60 (0.33–1.10)	0.101
Self-reported previous influenza vaccination		
No	1.00	
Yes	1.20 (0.55–2.63)	0.646
Employment outside the home		
No	1.00	
Yes, without anyone at work having symptoms of ARI during study period	**0.40 (0.22–0.72)**	**0.002**
Yes, with anyone at work having symptoms of ARI during study period	0.90 (0.44 –1.87)	0.784
Baseline antibody titer	**0.50 (0.28–0.87)**	**0.015**
No. household members, mean		
0–4 y	0.70 (0.45–1.10)	0.120
5–19 y	1.19 (0.97–1.45)	0.096
>19 y	**1.20 (0.99–1.44)**	**0.059**
Household contact with ARI symptoms during course of study		
No	1.00	
Yes	0.66 (0.39–1.14)	0.135
Travel out of Singapore during study period		
No	1.00	
Yes	**2.04 (1.28–3.25)**	**0.003**
Public transport		
Seldom	1.00	
Frequent	**1.73 (1.03–2.90)**	**0.040**
Hosmer-Lemeshow test for goodness-of-fit		**0.491**

[1]The multivariate model included all variables listed in the table. ARI, acute respiratory infection. **Boldface** indicates statistically significant association.
