# Supplementary material for: Risk Factors for Pandemic (H1N1) 2009 Seroconversion among Adults, Singapore, 2009
Source: Emerg Infect Dis. 2011 Aug;17(8):1455–62. doi: 10.3201/eid1708.101270 (PMC3381584; doi:10.3201/eid1708.101270)
Supplement: Related texts

# Research article: Risk Factors for Pandemic (H1N1) 2009 Seroconversion among Adults, Singapore, 2009

**DOI:** 10.3201/eid1708.101270

**Authors:** Wei-Yen Lim, Cynthia H.J. Chen, Yi Ma, Mark I.C. Chen, Vernon J.M. Lee, Alex R. Cook, Linda W.L. Tan, Norberto Flores Tabo, Ian Barr, Lin Cui, Raymond T.P. Lin, Yee Sin Leo, Kee Seng Chia

**Affiliations:** Author affiliations: National University of Singapore, Singapore (W.-Y. Lim, C.H.J. Chen, Y. Ma, M.I.C. Chen, V.J.M. Lee, L.W.L. Tan, N. Flores Tabo, Jr., K.S. Chia);; Tan Tock Seng Hospital, Singapore (M.I.C. Chen, Y.S. Leo);; Ministry of Defence, Singapore (V.J.M. Lee);; National University of Singapore, Singapore (A.R. Cook);; World Health Organization Collaborating Centre for Reference and Research on Influenza, Melbourne, Victoria, Australia (I. Barr);; Ministry of Health, Singapore (L. Cui, R.T.P. Lin)

## Abstract

A total of 828 community-dwelling adults were studied during the course of the pandemic (H1N1) 2009 outbreak in Singapore during June–September 2009. Baseline blood samples were obtained before the outbreak, and 2 additional samples were obtained during follow-up. Seroconversion was defined as a >4-fold increase in antibody titers to pandemic (H1N1) 2009, determined by using hemagglutination inhibition. Men were more likely than women to seroconvert (mean adjusted hazards ratio [HR] 2.23, mean 95% confidence interval [CI] 1.26–3.93); Malays were more likely than Chinese to seroconvert (HR 2.67, 95% CI 1.04–6.91). Travel outside Singapore during the study period was associated with seroconversion (HR 1.76, 95% CI 1.11–2.78) as was use of public transport (HR 1.81, 95% CI 1.05–3.09). High baseline antibody titers were associated with reduced seroconversion. This study suggests possible areas for intervention to reduce transmission during future influenza outbreaks.

Each year, influenza causes large numbers of deaths (*1**,**2*) and billions of dollars in direct medical costs and indirect costs from declines in productivity and worker absenteeism (*3*). Influenza vaccines help prevent some costs, but the uptake of vaccination for influenza varies widely (*4*), and effective vaccines may not be sufficient during influenza pandemics (*5*). Use of antiviral drugs to mitigate the effects of seasonal and pandemic influenza is also subject to such limitations as cost considerations (*6*), the need for adequate and timely delivery of antiviral drugs (*7*), and concerns about the emergence of resistance (*8*).

Nonpharmaceutical measures have been proposed as adjuncts for reducing the risk for influenza infection during pandemics and seasonal epidemics (*9*). Studies suggest that physical interventions, such as handwashing, use of protective equipment (e.g., face masks), and social distancing measures, can effectively reduce transmission of respiratory viruses, including influenza (*10**,**11*).

A novel influenza A virus, pandemic (H1N1) 2009 virus, emerged in Mexico and the United States during 2009 and spread worldwide within months (*12**,**13*). A few studies have investigated transmission of pandemic (H1N) 2009 virus (*14**,**15*) and the public health interventions that could be used to mitigate its spread (*16*). However, although some data are available from studies conducted in institutions and households (*17**,**18*), little is known about population-level risk factors for pandemic (H1N1) 2009 virus infection.

We investigated risk factors for serologically detected pandemic (H1N1) 2009 virus infection during the first wave of the epidemic in Singapore in 2009. Our study population was a prospective community-dwelling cohort of adults. Singapore is a tropical city-state and global travel hub in Southeast Asia with a population of 5.0 million persons. Singapore detected its first imported cases of pandemic (H1N1) 2009 in late May 2009 and subsequently experienced an epidemic wave lasting ≈12 weeks starting in late June, peaking during the first week of August, and subsiding by early September (*19**–**21*).

## Methods

### Study Design and Recruitment

This prospective community cohort study was part of a larger study to determine serologic conversion to pandemic (H1N1) 2009 virus in different populations (*22*). Community-dwelling adults 21–75 years of age were recruited from an existing cohort study. The Singapore Consortium of Cohort Studies is a long-term study conducted by the National University of Singapore to study gene–environment interactions in chronic diseases. In June 2009, ≈9,000 persons were participating in the Multi-ethnic Cohort, a subcohort of the Singapore Consortium of Cohort Studies. Participants for the Multi-ethnic Cohort were recruited through public outreach and referrals from existing cohort members. From among these 9,000 participants, we contacted 1,296 randomly selected participants, of whom 894 (69%) agreed to participate. All participants provided written consent, and the study received ethics review and approval from the Institutional Review Board of the National University of Singapore.

We obtained up to 3 blood samples from each participant. Banked blood samples were used for the baseline sample (sample 1); these samples were obtained during June 29, 2005–June 27, 2009, before widespread community transmission of pandemic (H1N1) 2009 virus in Singapore. Two additional blood samples were obtained: an intra-epidemic sample (sample 2) collected ≈4 weeks after the epidemic peaked (August 20–29, 2009) and a postepidemic sample (sample 3) collected ≈4 weeks after epidemic activity had subsided (October 6–11, 2009).

A baseline phone interview with a standardized questionnaire was conducted at recruitment, followed by interviews every 2 weeks throughout the epidemic period. We obtained the following information: sociodemographic and personal behavior, such as sex and ethnicity, history of vaccination for seasonal influenza, and smoking; measures of social interaction, including frequency of use of public transport, travel overseas, and extent of social mixing (e.g., visits to mass entertainment and sports venues); information about household size, ages of household members, and whether other persons in the household and workplace had symptoms of acute respiratory infections (ARIs); and new-onset respiratory and constitutional symptoms.

### Laboratory and Statistical Methods

Venous blood was obtained from participants, and serum was extracted on the same day. The hemagglutination-inhibition assay was performed according to standard protocols at the World Health Organization Collaborating Center for Reference and Research on Influenza in Melbourne, Victoria, Australia (*22*). We defined seroconversion as a >4-fold increase in antibody titers between any successive pairs of blood samples (between sample 1 and sample 2 for participants who provided only 1 other sample in addition to baseline; and between samples 1 and 2 or between samples 2 and 3 for participants who provided 2 samples in addition to baseline). We considered participants to have seroconverted in the few instances in which titers increased >4-fold between samples 1 and 2 but not between samples 1 and 3.

The primary outcome of interest was seroconversion, and we evaluated other variables as independent predictors for seroconversion. Sociodemographic and biologic factors analyzed were age (calculated from date of birth obtained from the National Registration Identity Card [NRIC]), sex (obtained from NRIC), ethnicity (the 3 major ethnic groups in Singapore—Chinese, Malay, and Indian—and a category for other minorities, obtained from NRIC), self-reported dwelling type, self-reported vaccination >1× previously with the seasonal influenza vaccine (ever vs. never vaccinated), baseline antibody titer to pandemic (H1N1) 2009, and smoking behavior (current, former, and never). Measures of social mixing were self-reported overseas travel, self-reported frequency of use of public transport (bus and the Mass Rapid Transit metro system), and self-reported frequency of visits to the following places: mass entertainment venues (e.g., cinemas, stadiums, and theaters; shopping centers, markets, and supermarkets; restaurants, bars, clubs, and other eating or drinking establishments; places of worship; and other social gatherings with >10 persons). Except for overseas travel, these measures reflected lifestyle routine, and the behavior reported in the baseline interview was used to reduce any effect from reverse causality (e.g., avoiding particular activities or locations because of influenza symptoms). For the overseas travel variable, participants were classified as having ever traveled during the entire study period. We also examined the effects of the number of contacts in various age groups living in the same household and of exposure to contacts who were sick with ARI either at home or at work.

Bivariate analyses were performed for all variables, with a final multivariate model constructed with the key sociodemographic variables of age, ethnicity, sex, dwelling type (as a proxy for socioeconomic status), and variables of interest to our study (smoking, previous influenza vaccination, baseline antibody titer, travel and public transport use, working outside the home, contact with other persons at home or at work who had ARI, and number of household members), by using a Weibull proportional hazards model. We chose to work with a parametric survival analysis model rather than with the more familiar Cox semiparametric proportional hazards model because 1) event (seroconversion) times were all interval- or right-censored (rather than the more common combination of uncensored and right-censored observations) and 2) as a result of the short period of blood sampling, there were many tied interval endpoints, making exact methods (such as that of Kalbfleisch and Prentice [*23*]) too exacting and approximations (such as those of Breslow [*24*] or Efron [*25*]) too approximate.

The study comprised participants from separate households and some from households where at least 1 other household member was also part of the study. Because influenza infection is transmissible, independence of outcomes cannot be assumed for persons from the same household. To reduce estimation errors, we randomly selected 1 person from each household for each regression analysis. We then repeated this selection process and analysis 1,000× to reduce the effect of chance variation resulting from the selection process. Reported hazard rates (HRs) refer to the mean HRs of all 1,000 iterations. Similarly, reported 95% confidence intervals (CIs) refer to the mean upper and lower 95% bounds from all 1,000 iterations. All p values reported are 2-tailed, with the significance level set at 0.05. We calculated goodness-of fit by using a modified version of the Hosmer-Lemeshow test (*26*). The statistical package R version 2.9.2 (*27*) was used for all analyses.

## Results

Of the 894 persons who agreed to participate in the study, 828 (93%) completed the baseline questionnaire. Baseline blood samples were available for all participants, sample 2 for 621 (69.5%) participants, and sample 3 for 689 (77.1%) participants. Three blood samples were available for 584 (65.3%) participants, and at least 2 samples were available for 727 (81.3%) persons. Figure 1 shows the pandemic (H1N1) 2009 epidemic curve in Singapore and the recruitment process for this study. When we compared characteristics of the 727 participants who completed the study with the 828 who were enrolled at the start of the study (Table), we found them to be broadly similar. All subsequent analyses were performed on these 727 persons. Of these, 494 came from separate households and 233 came from 117 households where at least 1 other household member was also part of this study; selection for 1 member per household resulted in a sample size of 611 persons.

**Figure 1:** A) Estimated pandemic (H1N1) 2009 curve, Singapore, June–September 2009. Data obtained from general practice sentinel data. Blue bars indicate number of influenza-like illness episodes per general practice per week. Red curve indicates estimated number of consults for pandemic (H1N1) 2009 per general practice per week. Orange bars indicate period of blood sample collection in the adult cohort (*23*). B) Summary of recruitment numbers and dates of blood collection in the adult community cohort.

**Table:** Comparison of participants who provided follow-up blood samples with cohort at start of study of risk factors for pandemic (H1N1) 2009 seroconversion, Singapore, 2009*

Characteristic	Total, n = 828	Respondents, n = 727
Age, y, mean (SD)	43.4 (12.0)	43.6 (11.8)
Age group, y		
20–29	149 (18.0)	124 (17.1)
30–39	131 (15.8)	113 (15.5)
40–49	285 (34.4)	260 (35.8)
50–59	175 (21.1)	155 (21.3)
>60	88 (10.6)	75 (10.3)
Sex		
F	482 (58.2)	432 (59.4)
M	346 (41.8)	295 (40.6)
Ethnicity		
Chinese	94 (11.4)	90 (12.4)
Malay	374 (45.2)	331 (45.5)
Indian	353 (42.6)	299 (41.1)
Other	7 (0.9)	7 (1.0)
Dwelling type		
<3-room public housing	202 (24.4)	177 (24.3)
4-room public housing	360 (43.5)	310 (42.6)
5-room public housing or private housing	266 (32.1)	240 (33.0)
Smoking		
Current smoker	181 (21.9)	149 (20.5)
Nonsmoker/former smoker	647 (78.1)	578 (79.5)
No. household members		
1	580 (70.1)	496 (68.2)
2	208 (25.1)	195 (26.8)
3	36 (4.4)	33 (4.5)
4	4 (0.5)	3 (0.4)
Self-reported previous influenza vaccination		
No	751 (90.7)	662 (91.1)
Yes	77 (9.3)	65 (8.9)
Employment outside the home		
No	308 (37.2)	277 (38.0)
Yes	520 (62.8)	450 (62.0)
Baseline antibody titer		
Mean (SD)	0.22 (0.71)	0.25 (0.75)
By age group, y		
20–29		0.48 (1.06)
30–39		0.19 (0.58)
40–49		0.17 (0.59)
50–59		0.28 (0.84)
>60		0.12 (0.52)
Public transport		
Seldom	294 (35.5)	249 (34.3)
Frequent	534 (64.5)	478 (65.7)

*Values are no. (%) except as indicated. Respondents are participants who provided at least 1 blood sample in addition to that obtained at baseline.

In bivariate analyses, seroconverters were younger, more likely to smoke, and more likely to have traveled outside Singapore than were nonseroconverters (Table A1). Seroconverters also were more likely to be of Malay ethnicity and to have more household members 5–19 years of age; however, p values for these 2 variables were just >0.05.

We assessed the frequency of activities and visits to a variety of public places, including use of public transport, during a 14-day period at baseline among the 727 participants and the proportion in each frequency group who seroconverted by the end of the study (Figure 2; Technical AppendixTable 1). The frequencies of activities and visits to public venues were not associated with seroconversion. More frequent use of public transport appeared to be associated with higher seroconversion rate. The proportion of seroconverters in the “never” and “once/twice” categories were similar, as were those in the “several times” and “daily/almost daily” categories. We dichotomized this variable into frequent use (i.e., participants reporting several times or daily/almost daily use of public transport during the preceding 14 days at baseline) and seldom use (participants reporting no use or use once or twice during the preceding 14 days. Frequent users of public transport were more likely to seroconvert; however, this finding was not statistically significant.

**Figure 2:** Study of seroconversion for pandemic (H1N1) 2009 virus, Singapore, June–September 2009. Public places were as follows: mass entertainment venues (e.g., cinemas, stadiums, and theaters; shopping centers, markets, and supermarkets; restaurants, bars, clubs, and other eating or drinking establishments; places of worship; and other social gatherings with >10 persons. Horizontal lines denote error bars for the estimates.

In multivariate analysis (Table A1), men were more likely than women to seroconvert (mean HR 2.23, 95% CI 1.26–3.93). Malays were more likely than Chinese to seroconvert (mean HR 2.67, 95% CI 1.04–6.91). Working outside the home and not having a work contact with ARI symptoms was associated with lower likelihood of seroconversion (mean HR 0.39, 95% CI 0.21–0.7); however, this decreased risk was not seen in workers who reported a work contact with ARI symptoms. Frequent use of public transport (mean HR 1.81, 95% CI 1.05–3.09) and travel out of the country (mean HR 1.76, 95% CI 1.11–2.78) also were associated with a higher risk for seroconversion, whereas high baseline antibody titers to pandemic (H1N1) 2009 virus were associated with lower risk for seroconversion (mean HR 0.5, 95% CI 0.27–0.94). The number of household members 5–19 years of age was marginally associated with increased risk for seroconversion (mean HR 1.17, 95% CI 0.95–1.43 for each additional member, compared with not having a household member in that age group), although the p value was not significant. There was good fit for the multivariate model (p = 0.625).

We repeated analyses (Technical Appendix Tables 2, 3) by using a logistic regression model for all 727 participants. Odds ratios (ORs) were broadly similar to the corresponding HRs when Weibull regression was used, with significant associations obtained for foreign travel, public transport use, high baseline antibody titers, sex, ethnicity and employment outside the home (Technical Appendix Tables 2, 3). These findings suggested that results are robust to the choice of modeling framework.

We investigated whether the association between seroconversion and foreign travel was greater in the earlier than in the later part of the outbreak by considering the subset of participants who provided 3 blood samples. We defined early seroconverters as participants who seroconverted between samples 1 and 2 and late seroconverters as those who converted between samples 2 and 3, and we investigated the estimated risk associated with foreign travel in logistic regression. After adjustment, the OR for foreign travel among early seroconverters was 2.06 (95% CI 1.25–3.55), whereas that for late seroconverters was 1.71 (95% CI 0.63–4.68).

## Discussion

Understanding the factors that influence the risk for influenza infection will permit development of rational response strategies to reduce transmission. Although studies have been conducted of household and institutional risk factors (*14**,**17**,**18**,**28*) for pandemic (H1N1) 2009 virus transmission, few data are available on risk factors in the general population. Through the use of a population-based cohort design, our study suggests that several factors are associated with pandemic (H1N1) 2009 infection.

A history of travel abroad was associated with increased risk for seroconversion. Self-reports obtained throughout the outbreak were used, and this result could have been subject to reverse causation; i.e., symptomatic persons may have reduced their frequency of travel. However, this bias, if present, would have resulted in a lower estimate of the true risk and therefore cannot explain the association observed. Whether the increased risk for seroconversion associated with overseas travel results from prolonged close proximity with infected travelers in airports and on airplanes or to transmission within other countries is unclear. Compared with the OR for early seroconverters, the OR for foreign travel in late seroconverters was not attenuated. If the risks associated with foreign travel resulted primarily from increased likelihood of transmission overseas, then we would expect the risk to be high during the start of an epidemic and drop substantially as local transmission increased. Our results imply that other explanations may be relevant, such as increased infection from prolonged close proximity with other infected persons during travel (e.g., on an airplane) or to increased use of public transport, such as buses or subway systems, during travel. A recent publication based on a retrospective cohort study of confirmed pandemic (H1N1) 2009 infection in persons returning from Mexico to New Zealand on a commercial airliner suggested a measureable risk associated with being on an airliner with infected persons (*29*). Further research is needed to understand the association between foreign travel and seroconversion. Although total restriction on global travel will not be feasible and is unlikely to prevent importation of infectious cases (*30*), such measures as travel advisories that promote personal hygiene and advise passengers to seek medical assistance promptly if symptoms develop might mitigate the effect of international travel on influenza transmission (*9**,**31**,**32*).

The local use of public transport was significantly associated with increased risk for seroconversion, even after adjustment. Confounding is unlikely to explain this association. Public transport in the local context involves close contact with a large number of persons, often for prolonged periods. Mathematical modeling studies have suggested that social distancing and reduction of mass gatherings are critical for preventing influenza spread (*9*). Although shutting down public transport systems is not possible without severe socioeconomic impact, other measures, such as reduction of crowding in public transport systems; better air circulation; and advice on personal hygiene etiquette and measures, including face mask use, may help to reduce transmission. Further studies are needed to characterize the mechanisms of influenza and respiratory virus transmission within public transport systems.

Compared with participants who did not have a job or worked from home, persons who worked away from home had a lower risk for seroconversion, but this risk was observed only among those who reported that no one in their workplace had symptoms of ARI. These findings suggest that transmission within the household may be more relevant than in the workplace. One possible explanation is that persons who do not work may be more involved in childcare activities at home. One study found that spending long periods exposed to a sick index patient is a risk factor for infection (*17*), and other studies have implicated children, especially those that are attending school, as a source of influenza transmission (*33**,**34*). Our study suggests that having a school-aged household member (5–19 years of age) increased risk for seroconversion (however, in multivariate analyses, the effect was attenuated and was not statistically significant). Interventions that target schoolchildren, such as vaccination and school closures, may reduce infection among other family members.

Household studies, such as that by Cauchemez et al. (*14*), have shown that increasing household size reduced the probability of transmission by each index patient. The amount of time spent by any 1 person in close contact with an index patient is most likely to be lower in larger households. However, such studies do not assess the aggregate risk when >1 household members become infected during an outbreak. Our study suggests that having more household members in the 5–19-year age group increases overall risk for seroconversion during a pandemic, a finding that is biologically plausible because every household member is at risk for infection (and subsequent transmission) during an outbreak.

Having a contact with ARI at home was not associated with increased risk for seroconversion. This finding might result from the low specificity and sensitivity of ARI as a marker for exposure to pandemic (H1N1) 2009 because other viral causes of ARI (including other influenza strains) were cocirculating in substantial proportion in Singapore during the pandemic (*19*). Infections also could have been transmitted by asymptomatic infected persons—indeed, 27% of persons in our study who seroconverted did not have any ARI symptoms throughout the study period.

Baseline antibody titers were associated with lower seroconversion rates. Older participants, especially those >60 years of age, appeared to have a lower risk for seroconversion (however, this finding was not statistically significant after multivariate adjustment). Other studies also have reported lower rates of infection among older persons (*35*). Some groups (*36*) have proposed that previously acquired immunity might explain the protection, although in our study, older participants did not have higher levels of measureable antibodies to pandemic (H1N1) 2009 virus. Other factors, such as lower levels of social contact among elderly persons, might explain the observation.

Men were more likely than women, and Malays were more likely than Chinese, to seroconvert. Although the association with male sex has not previously been reported, a study of the 1957 influenza pandemic in Singapore also suggested higher infection rates in Malays (*37*). Unmeasured sociocultural and behavioral factors might explain these observations, and further studies are needed to confirm these observations and to understand the basis for the association.

Our study did not find any effect of the frequency of visits to public venues on the risk for seroconversion (results not shown). We could not obtain accurate estimates of the length of time spent at each venue, the number of persons with whom each participant had contact during the visit, and the proximity and intensity of contact. Studies that capture such detail would be useful to quantify the relative risks for various activities and visits to different public venues during pandemics. Such a study would have public health policy implications because one option for social distancing is to close public venues, such as cinemas and theaters, an option taken by the authorities in Mexico during the early stages of the pandemic (*38*).

Our study had other limitations. We used data obtained at baseline because these would not be subject to reverse causation. However, behavior recorded at baseline might not reflect actual behavior during the outbreak. We were unable to study the effect of personal hygiene measures, such as use of face masks and handwashing, because of the complexity of measuring these factors by using questionnaires every 2 weeks.

Our prospective cohort study suggests avenues for further research into public health interventions for influenza epidemics. We showed that overseas travel and use of public transport were significantly associated with increased risk for seroconversion, and these offer potential areas for public health intervention, such as travel restriction, social distancing, and personal protection measures. Additional research is required to understand the reasons behind the increased risks associated with demographic factors of sex and ethnicity.

## Appendix

**Table A1:** Bivariate and multivariate analyses of factors associated with seroconversion to pandemic (H1N1) 2009 in a cohort of community-dwelling adults, Singapore, 2009*

Variable	Seroconverted, n = 98	Did not seroconvert, n = 629	Bivariate analysis		Multivariate analysis

Mean HR (95% CI)†	Mean p value‡		Mean HR (95% CI)†	Mean p value‡
Age, y							
Mean	41.14	44.05	0.79 (0.65–0.95)	0.034			
Median	43	45					
Range	21–64	21–82					
Age group, y							
20–29	21 (16.9)	103 (83.1)	1.00			1.00	
30–39	16 (14.2)	97 (85.8)	0.84 (0.42–1.67)	0.627		1.10 (0.52–2.36)	0.772
40–49	42 (16.2)	218 (83.8)	0.78 (0.44–1.39)	0.423		0.94 (0.46–1.94)	0.780
50–59	15 (9.7)	140 (90.3)	0.51 (0.25–1.03)	0.096		0.63 (0.29–1.34)	0.259
>60	4 (5.3)	71 (94.7)	0.25 (0.07–0.86)	0.054		0.31 (0.09–1.12)	0.105
Sex							
F	52 (12.0)	380 (88.0)	1.00			1.00	
M	46 (15.6)	249 (84.4)	1.48 (0.95–2.29)	0.128		**2.23 (1.26–3.93)**	**0.024**
Ethnicity							
Chinese	6 (6.7)	84 (93.3)	1.00			1.00	
Malay	56 (16.9)	275 (83.1)	**2.76 (1.14–6.69)**	**0.054**		**2.67 (1.04–6.91)**	**0.075**
Indian	34 (11.4)	265 (88.6)	1.55 (0.61–3.91)	0.390		1.94 (0.74–5.09)	0.218
Other	2 (28.6)	5 (71.4)	2.19 (0.26–18.54)	0.482		2.65 (0.30–23.52)	0.400
Dwelling type							
≤3-room public housing	21 (11.9)	156 (88.1)	1.00			1.00	
4-room public housing	51 (16.5)	259 (83.5)	1.58 (0.88–2.83)	0.173		1.39 (0.75–2.61)	0.348
5-room public housing or private housing	26 (10.8)	214 (89.2)	1.08 (0.57–2.06)	0.763		1.01 (0.50–2.05)	0.800
Smoking							
Current smoker	30 (20.1)	156 (79.1)	1.00			1.00	
Nonsmoker/former smoker	68 (11.8)	156 (88.1)	**0.52 (0.32–0.83)**	**0.027**		0.64 (0.35–1.14)	0.171
Household members							
1	63 (12.7)	433 (87.3)	1.00				
2	33 (16.9)	162 (83.1)	1.34 (0.78–2.29)	0.371			
3	2 (6.1)	31 (93.9)	0.89 (0.17–4.98)	0.666			
4	0	3 (100.0)		0.400			
Self-reported previous influenza vaccination							
No	88 (13.3)	574 (86.7)	1.00			1.00	
Yes	10 (15.4)	55 (84.6)	1.06 (0.51–2.22)	0.758		1.13 (0.51–2.48)	0.721
Employment outside the home							
No	42 (15.2)	235 (84.8)	1.00			1.00	
Yes, without anyone at work having ARI symptoms during study period	39 (11.2)	310 (88.8)	0.66 (0.41–1.08)	0.142		**0.39 (0.21–0.70)**	**0.013**
Yes, with anyone at work having ARI symptoms during study period	17 (16.8)	84 (83.2)	1.18 (0.65–2.17)	0.605		0.89 (0.44–1.80)	0.715
Baseline antibody titer							
Mean	0.082	0.272	0.54 (0.29–1.00)	0.086		**0.50 (0.27–0.94)**	**0.059**
Median	0	0					
No. household members 0–4 y							
Mean	0.204	0.291	0.79 (0.52–1.20)	0.287		0.71 (0.45–1.12)	0.166
Median	0	0					
Range	0–3	0–3					
No. household members 5–19 y							
Mean	1.57	1.22	**1.19 (1.02–1.40)**	**0.064**		1.17 (0.95–1.43)	0.170
Median	1.0	1					
Range	0–6	0–5					
No. household members >19 y							
Mean	3.33	3.10	1.14 (0.98–1.33)	0.131		1.13 (0.94–1.36)	0.216
Median	3	3					
Range	1–8	1–9					
Household contact with ARI symptoms during course of study							
No	72 (14.4)	428 (85.6)	1.00			1.00	
Yes	26 (11.5)	201 (88.5)	0.75 (0.46–1.22)	0.279		0.71 (0.41–1.21)	0.243
Travel out of Singapore during study period							
No	50 (10.2)	438 (89.8)	1.00			1.00	
Yes	48 (20.1)	191 (79.9)	**2.10 (1.35–3.25)**	**0.012**		**1.76 (1.11–2.78)**	**0.044**
Public transport							
Seldom	26 (10.4)	223 (89.6)	1.00			1.00	
Frequent	72 (15.1)	406 (84.9)	1.61 (0.96–2.70)	0.106		**1.81 (1.05–3.09)**	**0.062**

*Values are no. (%) except as indicated. Multivariate model included all variables listed; goodness-of-fit was 0.625. **Boldface** indicates significance. HR, hazard rate; CI, confidence interval; ARI, acute respiratory infection. †Calculated as the mean HR of 1,000 separate analyses, where each analysis comprised 610 persons from different households; 1 person was randomly selected from households that had >1 member in the study. The means of the upper and lower bound of the 95% CIs were computed for each variable. ‡The mean of the p estimate from the 1,000 iterations was computed for each variable.

## References

[1] Molinari NA, Ortega-Sanchez IR, Messonnier ML, Thompson WW, Wortley PM, Weintraub E, The annual impact of seasonal influenza in the US: measuring disease burden and costs. Vaccine. 2007;25:5086–96. 10.1016/j.vaccine.2007.03.04617544181

[2] Lee VJ, Yap J, Ong JB, Chan KP, Lin RT, Chan SP, Influenza excess mortality from 1950–2000 in tropical Singapore. PLoS ONE. 2009;4:e8096. 10.1371/journal.pone.000809619956611PMC2779490

[3] Szucs T. The socio-economic burden of influenza. J Antimicrob Chemother. 1999;44(Suppl. B):11–5. 10.1093/jac/44.suppl_2.1110877457

[4] Macroepidemiology of Influenza Vaccination (MIV) Study Group. The macro-epidemiology of influenza vaccination in 56 countries, 1997–2003. Vaccine. 2005;23:5133–43. 10.1016/j.vaccine.2005.06.01016039762

[5] Hessel L. European Vaccine Manufacturers (EVM) influenza Working Group. Pandemic influenza vaccines: meeting the supply, distribution and deployment challenges. Influenza Other Respir Viruses. 2009;3:165–70. 10.1111/j.1750-2659.2009.00085.x19627373PMC4634681

[6] Carrasco LR, Lee VJ, Chen MI, Matchar DB, Thompson JP, Cook AR. Strategies for antiviral stockpiling for future influenza pandemics: a global epidemic–economic perspective. J R Soc Interface. 2011 Feb 4; [Epub ahead of print].10.1098/rsif.2010.0715PMC314072121296791

[7] Arinaminpathy N, McLean AR. Antiviral treatment for the control of pandemic influenza: some logistical constraints. J R Soc Interface. 2008;5:545–53. 10.1098/rsif.2007.115217725972PMC3226978

[8] Janies DA, Voronkin IO, Studer J, Hardman J, Alexandrov BB, Treseder TW, Selection for resistance to oseltamivir in seasonal and pandemic H1N1 influenza and widespread co-circulation of the lineages. Int J Health Geogr. 2010;9:13. 10.1186/1476-072X-9-1320181276PMC2882220

[9] Lee VJ, Lye DC, Wilder-Smith A. Combination strategies for pandemic influenza response–a systematic review of mathematical modeling studies. BMC Med. 2009;7:76. 10.1186/1741-7015-7-7620003249PMC2797001

[10] Jefferson T, Del Mar C, Dooley L, Ferroni E, Al-Ansary LA, Bawazeer GA, Physical interventions to interrupt or reduce the spread of respiratory viruses: systematic review. BMJ. 2009;339:b3675. 10.1136/bmj.b367519773323PMC2749164

[11] Cowling BJ, Chan KH, Fang VJ, Cheng CK, Fung RO, Wai W, Facemasks and hand hygiene to prevent influenza transmission in households: a cluster randomized trial. Ann Intern Med. 2009;151:437–46.1965217210.7326/0003-4819-151-7-200910060-00142

[12] World Health Organization. Global Alert and Response (GAR). Influenza-like illness in the United States and Mexico (24 April 2009) [cited 2010 Jul 2]. http://www.who.int/csr/don/2009_04_24/en/index.html

[13] World Health Organization. Human infection with new influenza A (H1N1) virus: clinical observations from Mexico and other affected countries, May 2009. Wkly Epidemiol Rec. 2009;84:185–9.19462531

[14] Cauchemez S, Donnelly CA, Reed C, Ghani AC, Fraser C, Kent CK, Household transmission of 2009 pandemic influenza A (H1N1) virus in the United States. N Engl J Med. 2009;361:2619–27. 10.1056/NEJMoa090549820042753PMC3840270

[15] Lee VJ, Yap J, Cook AR, Chen MI, Tay JK, Tan BH, Oseltamivir ring prophylaxis for containment of 2009 H1N1 influenza outbreaks. N Engl J Med. 2010;362:2166–74. 10.1056/NEJMoa090848220558367

[16] Tang S, Xiao Y, Yang Y, Zhou Y, Wu J, Ma Z. Community-based measures for mitigating the 2009 H1N1 pandemic in China. PLoS ONE. 2010;5:e10911. 10.1371/journal.pone.001091120585565PMC2887838

[17] France AM, Jackson M, Schrag S, Lynch M, Zimmerman C, Biggerstaff M, Household transmission of 2009 influenza A (H1N1) virus after a school-based outbreak in New York City, April–May 2009. J Infect Dis. 2010;201:984–92. 10.1086/65114520187740

[18] Iuliano AD, Reed C, Guh A, Desai M, Dee DL, Kutty P, Notes from the field: outbreak of 2009 pandemic influenza A (H1N1) virus at a large public university in Delaware, April–May 2009. Clin Infect Dis. 2009;49:1811–20. 10.1086/64955519911964

[19] Leo YS, Lye DC, Barkham T, Krishnan P, Seow E, Chow A. Pandemic (H1N1) 2009 surveillance and prevalence of seasonal influenza, Singapore. Emerg Infect Dis. 2010;16:103–5. 10.3201/eid1601.09116420031051PMC2874379

[20] Cutter J, Ang LW, Lai F, Subramony H, Ma S, James L. Outbreak of pandemic influenza A (H1N1–2009) in Singapore, May–September 2009. Ann Acad Med Singapore. 2010;39:273–10.20473451

[21] Ong JB, Chen MI-C, Cook A, Lee HC, Lee VJ, Lin RT, Real-time epidemic monitoring and forecasting of H1N1–2009 using influenza-like-illness from general practice and family doctor clinics in Singapore. PLoS ONE. 2010;5:e10036. 10.1371/journal.pone.001003620418945PMC2854682

[22] Chen MI, Lee VJM, Lim WY, Barr IG, Lin RT, Koh GC, 2009 Influenza A(H1N1) seroconversion rates and risk factors among distinct adult cohorts in Singapore. JAMA. 2010;303:1383–91. 10.1001/jama.2010.40420388894

[23] Kalbfleisch JD, Prentice RL. The statistical analysis of failure time data. 2nd ed. New York: Wiley; 2002.

[24] Breslow N. A generalized Kruskal-Wallis test for comparing κ samples subject to unequal patterns of censorship. Biometrika. 1970;57:579–94. 10.1093/biomet/57.3.579

[25] Efron B. The efficiency of Cox’s partial likelihood function for censored data. J Am Stat Assoc. 1977;72:557–65. 10.2307/2286217

[26] Hosmer DW, Lemeshow S. Applied logistic regression. 2nd ed. New York: John Wiley & Sons; 2000. p. 143–202.

[27] R Development Core Team. R: a language and environment for statistical computing. Vienna (Austria): R Foundation for Statistical Computing: 2010 [cited 2009 Oct 20]. http://www.r-project.org

[28] Lee VJ, Yap J, Cook AR, Chen MI, Tay JK, Barr IG, Effectiveness of public health measures in mitigating pandemic influenza spread: a prospective serological cohort study. J Infect Dis.10.1086/65648020863233

[29] Baker MG, Thornley CN, Mills C, Roberts S, Perera S, Peters J, Transmission of pandemic A/H1N1 2009 influenza on passenger aircraft: retrospective cohort study. BMJ. 2010;340:c2424. 10.1136/bmj.c242420495017PMC2874661

[30] Mukherjee P, Lim PL, Chow A, Barkham T, Seow E, Win MK, Epidemiology of travel-associated pandemic (H1N1) 2009 infection in 116 patients, Singapore. Emerg Infect Dis. 2010;16:21–6. 10.3201/eid1601.09137620031038PMC2874386

[31] Cooper BS, Pitman RJ, Edmunds WJ, Gay NJ. Delaying the international spread of pandemic influenza. PLoS Med. 2006;3:e212. 10.1371/journal.pmed.003021216640458PMC1450020

[32] Epstein JM, Goedecke DM, Yu F, Morris RJ, Wagener DK, Bobashev GV. Controlling pandemic flu: the value of international air travel restrictions. PLoS ONE. 2007;2:e401. 10.1371/journal.pone.000040117476323PMC1855004

[33] Monto AS, Davenport FM, Napier JA, Francis T Jr. Effect of vaccination of a school-age population upon the course of an A2-Hong Kong influenza epidemic. Bull World Health Organ. 1969;41:537–42.5309469PMC2427727

[34] Loeb M, Russell ML, Moss L, Fonseca K, Fox J, Earn DJ, Effect of influenza vaccination of children on infection rates in Hutterite communities: a randomized trial. JAMA. 2010;303:943–50. 10.1001/jama.2010.25020215608

[35] Fisman DN, Savage R, Gubbay J, Achonu C, Akwar H, Farrell DJ, Older age and a reduced likelihood of 2009 H1N1 virus infection. N Engl J Med. 2009;361:2000–1. 10.1056/NEJMc090725619907052

[36] Huang DT, Shao PL, Huang KL, Lu CY, Wang JR, Shih SR, Serologic status for pandemic (H1N1) 2009 virus, Taiwan. Emerg Infect Dis. 2011;17:76–8.2119285810.3201/eid1701.100014PMC3204618

[37] Lim KA, Smith A, Hale JH, Glass J. Influenza outbreak in Singapore. Lancet. 1957;273:791–6. 10.1016/S0140-6736(57)90893-013476727

[38] British Broadcasting Corporation News. Mexico shuts down to control flu (1 May 2009) [cited 2010 Jul 2].

# Research article: Risk Factors for Pandemic (H1N1) 2009 Seroconversion among Adults, Singapore, 2009

**DOI:** 10.3201/eid1708.101270

**Authors:** Wei-Yen Lim, Cynthia H.J. Chen, Yi Ma, Mark I.C. Chen, Vernon J.M. Lee, Alex R. Cook, Linda W.L. Tan, Norberto Flores Tabo, Ian Barr, Lin Cui, Raymond T.P. Lin, Yee Sin Leo, Kee Seng Chia

**Affiliations:** Author affiliations: National University of Singapore, Singapore (W.-Y. Lim, C.H.J. Chen, Y. Ma, M.I.C. Chen, V.J.M. Lee, L.W.L. Tan, N. Flores Tabo, Jr., K.S. Chia);; Tan Tock Seng Hospital, Singapore (M.I.C. Chen, Y.S. Leo);; Ministry of Defence, Singapore (V.J.M. Lee);; National University of Singapore, Singapore (A.R. Cook);; World Health Organization Collaborating Centre for Reference and Research on Influenza, Melbourne, Victoria, Australia (I. Barr);; Ministry of Health, Singapore (L. Cui, R.T.P. Lin)

## Earning CME Credit

To obtain credit, you should first read the journal article. After reading the article, you should be able to answer the following, related, multiple-choice questions. To complete the questions (with a minimum 70% passing score) and earn continuing medical education (CME) credit, please go to www.medscape.org/journal/eid. Credit cannot be obtained for tests completed on paper, although you may use the worksheet below to keep a record of your answers. You must be a registered user on Medscape.org. If you are not registered on Medscape.org, please click on the New Users: Free Registration link on the left hand side of the website to register. Only one answer is correct for each question. Once you successfully answer all post-test questions you will be able to view and/or print your certificate. For questions regarding the content of this activity, contact the accredited provider, CME@medscape.net. For technical assistance, contact CME@webmd.net. American Medical Association’s Physician’s Recognition Award (AMA PRA) credits are accepted in the US as evidence of participation in CME activities. For further information on this award, please refer to http://www.ama-assn.org/ama/pub/category/2922.html. The AMA has determined that physicians not licensed in the US who participate in this CME activity are eligible for *AMA PRA Category 1 Credits*™. Through agreements that the AMA has made with agencies in some countries, AMA PRA credit may be acceptable as evidence of participation in CME activities. If you are not licensed in the US, please complete the questions online, print the certificate and present it to your national medical association for review.

## Article Title: Risk Factors for Pandemic (H1N1) 2009 Seroconversion among Adults, Singapore, 2009

## CME Questions

**1. You are a member of a public health task force with the assignment to reduce the impact of influenza on your community. The task force director asks you to investigate risk factors for infection with pandemic (H1N1) 2009.Which of the following demographic variables was associated with a higher risk for pandemic (H1N1) 2009 seroconversion?**A. Age between 20 and 29 yearsB. Age older than 60 yearsC. Male sexD. 3-room or smaller dwelling**2. Which of the following factors most likely reduced the risk for pandemic (H1N1) 2009 seroconversion?**A. Malay ethnicityB. More international travelC. Working outside of the homeD. Regular tea consumption**3. Which of the following public health interventions may be most effective in preventing the spread of pandemic (H1N1) 2009?**A. Limiting theater seating to every other seatB. Closing restaurantsC. Improving air circulation on public transportationD. Limiting seating at places of worship**4. Which of the following laboratory values was associated with a reduced rate of pandemic (H1N1) 2009 seroconversion?**A. High baseline antibody titers to pandemic (H1N1) 2009B. Low baseline antibody titers to pandemic (H1N1) 2009C. Normal serum glucose levelsD. High serum white blood cell counts

## Activity Evaluation

**Table:** 

1. The activity supported the learning objectives.				
Strongly Disagree				Strongly Agree
1	2	3	4	5
2. The material was organized clearly for learning to occur.				
Strongly Disagree				Strongly Agree
1	2	3	4	5
3. The content learned from this activity will impact my practice.				
Strongly Disagree				Strongly Agree
1	2	3	4	5
4. The activity was presented objectively and free of commercial bias.				
Strongly Disagree				Strongly Agree
1	2	3	4	5

## Supplementary Material

Technical Appendix Baseline measures of social contact and interaction, Bivariate logistic regression, and Multivariate logistic regression analysis tables.
